# From Pure Tones to Complex Sounds: Expanding Audiology Tools to Better Address Speech and Language Development

**DOI:** 10.7759/cureus.72519

**Published:** 2024-10-28

**Authors:** Paris Binos, George Korres, Theodora Papastefanou, Nikolaos Papadimitriou, George Psillas

**Affiliations:** 1 CIRCLE Laboratory, Department of Rehabilitation Sciences, Cyprus University of Technology, Limassol, CYP; 2 2nd Otolaryngology Department, National and Kapodistrian University of Athens School of Medicine, Athens, GRC; 3 Department of Rehabilitation Sciences, Cyprus University of Technology, Limassol, CYP; 4 2nd ENT Department, Attikon University Hospital, National and Kapodistrian University of Athens, Athens, GRC; 5 1st Otolaryngology Department, School of Medicine, Aristotle University of Thessaloniki, Thessaloniki, GRC

**Keywords:** audiogram, audiological tests, pediatric hearing loss, speech language pathology, speech perception

## Abstract

This editorial highlights the limitations of relying solely on pure-tone audiometry for diagnosing and managing hearing loss, particularly in the fields of speech-language pathology and audiology. While pure-tone audiometry has long been the gold standard for assessing hearing sensitivity, its capacity to fully capture the complexities of hearing impairments is increasingly called into question. The article examines the profound impact of hearing loss on language development, psychosocial well-being, and quality of life, especially in infants and toddlers, who are at risk of significant delays in speech and language development. These delays affect various linguistic domains, including morphology, vocabulary, syntax, semantics, and speech intelligibility. Hearing loss often distorts sound perception, particularly of softer consonants and key morphemes critical for understanding verb tenses, possessives, and plurals, further hindering language comprehension and communication.

The article critiques traditional training programs for speech-language pathologists (SLPs) and audiologists, which tend to focus on basic pure-tone audiograms and standardized hearing loss classifications. It argues for a more comprehensive educational approach that emphasizes deeper audiogram interpretation, enabling improved diagnosis and management of hearing loss. Additionally, while remaining focused on pure-tone audiometry, the article discusses how SLPs can be better equipped to "decode" audiogram data, thereby enhancing early intervention strategies to support optimal language development in young children with hearing loss.

## Editorial

This editorial addresses the research question posed by Binos and Psillas (2024) to the Balkan ENT-HNS concerning the possible need for training beyond pure tones and the degree of hearing loss [1]. Pure-tone audiometry has been the primary tool for measuring hearing sensitivity and identifying various degrees of hearing loss for a long time [2]. However, many experts now realize that this method alone may not be sufficient to fully capture the complexities of hearing impairments and meet the diverse needs of patients, especially in the fields of speech-language pathology and audiology [3]. Hearing is an essential sensory modality that plays a critical role in language and communication development [4]. World Report on Hearing highlights that over 1.5 billion people globally experience declining hearing capacity, and at least 430 million will require care [5].

Hearing loss can significantly impact language development, psychosocial well-being, quality of life, educational attainment, and economic independence if not identified and addressed adequately. The World Health Organization estimates that more than one billion young people put themselves at risk of permanent hearing loss. Public health action is essential to addressing hearing loss and mitigating such risks. Infants and toddlers with hearing loss are at a higher risk for delayed speech and language development compared to typical developing children [6]. Some of the areas that are impacted, depending on the child, include disorders of morphology, vocabulary, syntax, semantics, and speech intelligibility. For example, a child diagnosed with moderately severe hearing loss has speech production errors comparable to those of typically developing children with phonological or articulation delays [7].

The degree of hearing loss impacts our understanding and comprehension of speech and language, including missing anywhere from 25% to 40% of the speech signal with a hearing loss of 40 dB HL [8]. Moderate hearing loss can be observed in a child whose air conduction thresholds fall within 41-55 dB HL. As a result, this individual misses as much as 80% of speech signals when verbally communicating [9]. On the other hand, children experiencing high-frequency sensorineural hearing loss can face even more significant challenges (downward-sloping audiograms), even with a slight loss of hearing. This condition notably hinders their ability to process speech, comprehend language, communicate effectively, learn in classroom settings, and develop socially.

Hearing loss typically results in a distortion or muffling of sounds, complicating the child's ability to understand subtle elements of spoken language. This mainly affects the perception of softer consonants, compared to vowels, like /f/, /s/, /sh/, /h/, and stop consonants including /p/, /t/, /k/, as well as critical morphemes that denote verb tenses, possessives, and plurals, such as "-ed," "'s," and "-s" [10]. It should be noted that the human ear is most sensitive to sounds in the mid-range of frequencies 1-2 KHz and poorer at the lower and higher frequencies. There is substantial disagreement among clinicians and researchers regarding the sound frequencies in predicting the speech recognition threshold (SRT). Despite the old theories that highlighted the importance of higher frequencies [11] in audiograms, more recent approaches support that even the frequency of 4K Hz, together with 500, 1K, and 2K Hz, efficiently represent the “real” SRT [12].

Training programs for speech-language pathologists (SLPs) and audiologists primarily focus on established methods, such as pure-tone audiograms and the American Speech-Language-Hearing Association's (ASHA) hearing loss classification system [13]. While these approaches are essential, they have significant limitations in thoroughly evaluating speech science and auditory processing. Also, these conventional tools often do not provide an adequate framework for effectively counseling individuals with different levels of hearing loss. According to ASHA, early identification and intervention are crucial for optimal language development in infants and toddlers with hearing loss [14]. SLPs are considered experts in treating and managing communication and swallowing skills across the lifespan. Thus, speech-language therapy (SLT) is an essential intervention for infants and toddlers with hearing loss, which can support their speech and language development and improve their communication skills [15]. SLT is a complex and multidisciplinary field that requires specialized knowledge and skills [16]. One of the critical skills that SLPs need to have as clinicians is the ability to "decode" an audiogram accurately [17,18] beyond the audiological information that it offers.

Thus, the pure-tone audiogram, though fundamental to audiology and rehabilitative audiology, has limitations, especially in speech science and auditory processing. An audiogram is a graph that displays the softest sounds an individual can hear at different frequencies [18,19]. Despite the audiological limitations, it is a critical tool for diagnosing hearing loss and designing appropriate intervention plans for infants and toddlers with hearing loss [20]. For example, cochlear damage does not necessarily result in abnormal pure-tone hearing tests, and individuals with acoustic neuromas exhibit standard hearing test results [21]. In contrast, the audiogram has some limitations when determining hearing loss involving the central auditory nervous system (CANS). Recent advancements in auditory neuroscience have revealed the key role of CANS in hearing and related disorders. While the audiogram helps determine the type, degree, and configuration of hearing loss, it only provides information about hearing sensitivity. It cannot assess, for example, central auditory processing or the processing of real-world signals like speech or music.

This article reviews the limitations of the pure-tone audiogram and offers insights into using behavioral audiological tests and electrophysiological procedures in conjunction with it to provide better diagnostic and rehabilitative information to clinicians and patients [22]. The purpose of this work is not to describe the significance or use of other audiometric equipment or techniques. Instead, it aims to help SLPs understand the information an audiogram provides concerning the speech perception skills of infants and toddlers with hearing loss and to emphasize the importance of being able to “decode” audiograms in SLP by discussing the strengths and limitations of using a pure-tone audiogram.

In audiology and SLP, the routine use of the pure-tone audiogram presents a complex challenge. Commonly perceived as a 'simple' test, the audiogram is far from a straightforward "functional audiogram." Its interpretation and associated implications should be understood more by those most affected by its outcomes - the hearing aid recipients. Even though almost every individual who receives a hearing aid undergoes this test, a comprehensive understanding of the audiogram results and their implications still needs to be obtained. This issue is compounded when prospective hearing aid users, already overwhelmed with new information, fixate on their test outcomes and the financial burden of hearing aids. In such scenarios, critical explanations provided by professionals are prone to be forgotten or misinterpreted in the subsequent weeks. As a result, SLPs and audiologists frequently encounter patients with misconceptions about what their audiogram results signify, revealing a gap between professional communication and patient understanding.

We should consider more effective communication strategies. The complexity of the pure-tone audiogram test is often underestimated, both by patients and, occasionally, by health professionals. While it is a standard procedure for those receiving hearing aids, the nuanced interpretation of its results and what they mean for the individual's auditory capabilities can be perplexing. Patients already grappling with the emotional impact of hearing loss and the financial considerations related to hearing aids can quickly become fixated on the raw outcomes of the test. This fixation may lead to the critical information provided by audiologists and SLPs needing to be remembered or understood over time. Misconceptions about audiogram results demonstrate an apparent disconnect between what the professional conveys and what the patient understands.

This gap reflects the complexity of interpreting audiogram results and underscores the need for more effective communication strategies to ensure patients accurately grasp the implications of their hearing assessment. Even if the miscommunication is unintentional, the consequence is the same: individuals misunderstand their hearing health and the impact of their audiograms. Therefore, finding more effective ways to communicate these results is crucial. Several strategies could help. The suggested strategies for enhancing patient understanding include:

1. Using analogies and visual aids to simplify complex audiogram data.

2. Implementing follow-up consultations to reinforce information and address new questions.

3. Providing personalized narrative summaries connecting audiogram outcomes to the patient's daily life.

4. Utilizing diverse communication methods to cater to different learning styles.

5. Involving real-life assessment questionnaires of hearing skills based on speech-in-competition tests.

6. Involving communication partners in the consultation for additional support.

7. Encouraging interactive sessions to foster engagement and better understanding.

Accordingly, using analogies and visual aids can help translate the abstract data of an audiogram into more tangible concepts. Another strategy involves the implementation of follow-up consultations, which can reinforce the initial information provided and address any questions that may have arisen after the patient has had time to process their results. Moreover, personalized narrative summaries that relate the audiogram outcomes to the patient's daily life activities can make the information more relatable and memorable. Educating patients on using various communication methods, such as written, verbal, and digital media, can cater to different learning styles and preferences, ensuring that the message is received and understood.

It is also beneficial to involve family members or close friends in the consultation process, as they can provide support and help remember the information provided. Interactive sessions, where patients are encouraged to ask questions and discuss their audiogram results, can foster a better understanding. The goal is to move from a one-size-fits-all approach to a more tailored communication method that takes into account the individual's circumstances, concerns, and comprehension levels. By doing so, we can empower patients with a clearer understanding of their hearing health and the implications of their audiogram, enabling them to make more informed decisions about their hearing aid use and overall auditory care.

Beyond the "traditional" audiological information

To enhance patient comprehension and engagement in their hearing health journey, we recommend adopting clearer, more accessible language accompanied by visual aids in the process of explaining various aspects of the audiogram. These aids can make explaining audiogram results more tangible and easier to understand. Additionally, scheduling a follow-up consultation a few weeks after the initial appointment could be highly beneficial. This follow-up session would provide an opportunity to revisit and clarify any misunderstandings about the audiogram results and address any new questions that may have emerged.

An effective habilitation program should include "reading" the audiogram, which requires specialized knowledge of the SLPs [14,17,21,23]. SLPs must have specialized training and expertise to interpret audiograms accurately and effectively [21,24]. They must evaluate the information obtained from an audiogram to design individualized intervention plans that support infants’ and toddlers' speech and language development. Thus, "reading" an audiogram offers a wealth of information about hearing loss and hard-of-hearing infants and toddlers.

An audiogram provides the “traditional” audiological information about the frequency (Hz), or pitch depicted on the horizontal axis, from low frequencies on the left (250 Hz) to high frequencies on the right (8,000 Hz), volume (dB), and minimum sound detection level (threshold) [19]. The correlation between frequency and pitch is directly proportional, albeit not linear. The sensitivity of the human auditory system varies across different frequency ranges. In frequencies below <1000 Hz, pitch perception demonstrates a relatively linear relationship with frequency. However, at higher frequencies, a significantly more significant frequency alteration is required to elicit a perceptible change in pitch. Intriguingly, in cases where a harmonic series’ fundamental frequency (f0) is absent, the auditory system exhibits a compensatory mechanism, allowing for the perception of the fundamental frequency despite the lack of lower harmonics [25].

Thus, the values of the first three formants (F) are essential for SLPs, as they provide information about the acoustic characteristics of speech sounds, especially vowel recognition [26]. SLPs also need to consider the values of the Ling Six Sound Test, which are critical for assessing the audibility of speech sounds [27]. In addition, the suprasegmental prosodic features such as duration (sec), volume (dB), pitch (f0) (related to the melody of music below the 1.5 KHz), and the acoustic detection of vowels (Vs) (audible below the 1.5 KHz), consonants (Cs) (nasals are audible below the 1.5 KHz while the majority of acoustic energy of consonants above the 1.5 KHz) are also essential aspects of "reading" an audiogram [28]. An audiogram provides information about the magnitude of the hearing loss per frequency and the range of speech frequencies necessary for the perception of speech [29]. The changes in the oral cavity's shape and size and the nasal joint can affect the above values [30]. Therefore, SLPs must consider these factors when evaluating and designing individualized intervention plans for hard-of-hearing infants and toddlers.

A unique role in "reading" an SLP should convey is the average of the four-frequency tone audiogram (PTA), as it provides quantitative information on the auditory sensitivity of speech recognition [23]. In 1946, Carhart studied this relationship between pure-tone thresholds at 500, 1K, and 2K Hz and the speech reception threshold that linked which type of curve, duration, and type of hearing loss interfered with this relationship [31]. The PTA calculates the average hearing threshold at four different frequencies, including 500 Hz, 1000 Hz, 2000 Hz, and 4000 Hz [32]. Hence, the impact of hearing loss on speech perception is better based on the mean square error that has identified more significant errors when using thresholds of 500, 1000, and 2000 Hz, while analyses based on linear regression showed a higher correlation (91%) between the four frequencies instead of three (88%) [12].

Based on PTA, the famous “speech banana audiograms” present an otherwise static visual representation of where various speech sounds fall on the audiogram. However, this representation does not fully capture the dynamic nature of real-life conversations, where speech sounds, or phonemes, occur rapidly and are influenced by the surrounding sounds within a spoken phrase. These surrounding sounds partly shape the acoustic characteristics of speech, and the interaction of these sounds with the suprasegmental features of speech signals can significantly alter how vowels are perceived concerning preceding and following consonants. These complex interactions provide additional cues crucial for understanding speech and may explain why some individuals understand speech better than what their audiogram results indicate.

Despite its limitations, the "speech banana" audiogram offers a foundational guide to the typical positions of speech sounds, which is invaluable for understanding the implications of different types of hearing loss. When the audiogram is analyzed concerning the "speech banana," it becomes evident how specific hearing loss affects the perception of speech sounds differently. For instance, those with specific hearing losses may find lower-frequency sounds more discernible than higher-frequency ones, potentially struggling to hear sounds like the /s/ sound. This understanding is critical to assessing and addressing the unique challenges individuals with hearing impairments face.

Audiograms, the cornerstone of audiology practices, can become so ingrained in an SLP’s daily routine that they might unconsciously condense and simplify their explanation, particularly after innumerable repetitions [33]. This habitual recitation, primarily driven by memory, may overlook that the patient may not comprehend the information as intended. For patients, many of whom might be encountering terms like "pure tone audiometer" for the first time in the audiologist’s office, the complexity of understanding this information is rather significant [34]. Consequently, it becomes unrealistic to expect patients to immediately grasp the intricate connections between their specific hearing loss pattern and the subsequent impact on their listening habits [35]. As such, SLPs must devise strategies to improve the delivery and comprehension of this crucial information [36].

"Decoding" an audiogram is an essential clinical skill for SLPs working with hard-of-hearing infants and toddlers. The information obtained from an audiogram is critical for designing individualized intervention plans, identifying the fundamental frequencies and sounds vital for speech perception, and monitoring progress. However, the studies also reveal that interpreting audiograms in this population poses unique challenges and limitations that must be addressed. Therefore, SLPs must receive specialized training to accurately interpret audiograms and provide the best possible care for these young children. Future research could investigate the efficacy of incorporating other tools and approaches, such as electrophysiological measures, to supplement audiograms and obtain a more comprehensive assessment of hearing function in hard-of-hearing infants and toddlers. Also, we recommend more extensive studies with standardized protocols and outcome measures to improve the consistency and accuracy of audiogram interpretation and to guide evidence-based clinical practice (EBP). By addressing these challenges, SLPs can provide optimal rehabilitation programs to improve the quality of life and promote successful language development in hard-of-hearing infants and toddlers.

A fundamental competency for SLPs constitutes the precise interpretation of audiograms. This skill is indispensable for crafting tailored intervention strategies that meet the unique needs of hard-of-hearing children. An audiogram reveals the extent of hearing loss across different frequencies but identifies the specific speech frequencies critical for speech perception as well. In their evaluations and planning of individualized interventions, SLPs must rely on audiological information, while trying to "decode" the developmental changes in the oral cavity's and nasal passage's shape and size, ensuring that all children receive the most effective support to thrive in their communication journey.

## References

[1] Binos P, Psillas G (2024). Binos P, Psillas G: audiogram: is there a need to get trained beyond pure tones and the degree of hearing loss?. https://www.researchgate.net/publication/384386484_Audiogram_Is_There_a_Need_to_Get_Trained_Beyond_Pure_Tones_and_the_Degree_of_Hearing_Loss/citation/download.

[2] Goodwin MV, Hogervorst E, Maidment DW (2024). Hearing difficulties and memory problems: the mediating role of physical health and psychosocial wellbeing. Int J Audiol.

[3] Iliadou VM, Bamiou DE, Keith W, Purdy SC, Thai-Van H (2024). It is time to change the way we think about hearing evaluation. Eur Arch Otorhinolaryngol.

[4] Moeller MP (2000). Early intervention and language development in children who are deaf and hard of hearing. Pediatrics.

[5] Chadha S, Kamenov K, Cieza A (2021). The world report on hearing, 2021. Bull World Health Organ.

[6] Korver AM, Smith RJ, Van Camp G (2017). Congenital hearing loss. Nat Rev Dis Primers.

[7] Show RL, Nerbonne MA (2007). Introduction to Audiologic Rehabilitation (5th ed.).

[8] Anderson K., Matkin N (2024). Relationship of long term hearing loss to psychosocial impact and educational needs. https://www.wyomingehdi.org/wp-content/uploads/2012/04/Relationship-of-Hearing-Loss-to-Listening-and-Learning-Needs.pdf.

[9] Welling D, Ukstins C (2017). Fundamentals of Audiology for the Speech-Language Pathologist (2nd). https://books.google.com.cy/books/about/Fundamentals_of_Audiology_for_the_Speech.html?id=egZVAgAAQBAJ&redir_esc=y.

[10] Levey S, Fligor BJH, Ginocchi C, Kagimbi L (2012). The effects of noise-induced hearing loss on children and young adults. Contemp Issues Commun Sci Disord.

[11] Kryter KD, Williams C, Green DM (1962). Auditory acuity and the perception of speech. J Acoust Soc Am.

[12] Andrade KC, Menezes Pde L, Carnaúba AT, Rodrigues RG, Leal Mde C, Pereira LD (2013). Non-flat audiograms in sensorineural hearing loss and speech perception. Clinics (Sao Paulo).

[13] (2024). ASHA, American Speech-Language-Hearing Association. Type, degree, and configuration of hearing loss. https://www.asha.org/siteassets/ais/ais-type-degree-and-configuration-of-hearing-loss.pdf.

[14] (2024). ASHA Association. Guidelines for audiologic screening. https://www.iup.edu/special-ed/files/programs/guidelines-for-audiologic-screening.pdf.

[15] Ertmer DJ, Mellon JA (2001). Beginning to talk at 20 months: early vocal development in a young cochlear implant recipient. J Speech Lang Hear Res.

[16] Geers AE, Brenner C, Tobey EA (2011). Article 1: Long-term outcomes of cochlear implantation in early childhood: sample characteristics and data collection methods. Ear Hear.

[17] ASHA Association (2024). ASHA Association: report of the joint coordinating committee on evidence-based practice. https://www.asha.org/siteassets/uploadedfiles/jccebpreport04.pdf.

[18] Gaspard JC, Bauer GB, Reep RL, Dziuk K, Cardwell A, Read L, Mann DA (2012). Audiogram and auditory critical ratios of two Florida manatees (Trichechus manatus latirostris). J Exp Biol.

[19] (2024). ASHA Association. Audiograms. https://www.asha.org/public/hearing/audiogram/.

[20] Bess FH, Dodd-Murphy J, Parker RA (1998). Children with minimal sensorineural hearing loss: prevalence, educational performance, and functional status. Ear Hear.

[21] Beck HJ, Beatty CW, Harner SG, Ilstrup DM (1986). Acoustic neuromas with normal pure tone hearing levels. Otolaryngol Head Neck Surg.

[22] Musiek FE, Shinn J, Chermak GD, Bamiou DE (2017). Perspectives on the pure-tone audiogram. J Am Acad Audiol.

[23] Sommerfeldt J, Kolb CM (2023). Hearing Loss Assessment in Children. https://www.ncbi.nlm.nih.gov/books/NBK580492/.

[24] ASHA Association (2024). ASHA Association: scope of practice in speech-language pathology. https://www.asha.org/policy/sp2016-00343/.

[25] Raphael L, Borden G, Harris K (2011). Speech Science Primer (6th ed.). https://shop.lww.com/Speech-Science-Primer/p/9781608313570.

[26] Kent RD, Vorperian HK (2013). Speech impairment in Down syndrome: a review. J Speech Lang Hear Res.

[27] Ling D (1978). Speech development in hearing-impaired children. J Commun Disord.

[28] Krishnan G, Pujari R, Roy K (2020). Literacy-based normative data for elderly adults on linguistic profile test in Kannada and Malayalam. Ann Indian Acad Neurol.

[29] Northern JL, Downs MP (2002). Hearing in Children. https://books.google.com.cy/books/about/Hearing_in_Children.html?id=1CUFXsBbBgoC&redir_esc=y.

[30] Swanepoel de W, Hall JW 3rd (2010). A systematic review of telehealth applications in audiology. Telemed J E Health.

[31] Carhart R (1946). Individual differences in hearing for speech. Ann Otol Rhinol Laryngol.

[32] Kaplan H, Gladstone VS, Lloyd LL (1993). Audiometric Interpretation: A Manual of Basic Audiometry (2nd ed). Audiometric Interpretation: A Manual of Basic Audiometry (2nd ed).

[33] Racey A, Riess M (2024). Racey A, Riess M: bridging the gap: shifting an audiology practice to include implant services. https://hearingreview.com/practice-building/bridging-gap-shifting-audiology-practice-include-implant-services.

[34] Hendler CB (2024). Hendler CB: 10 tactics to improve patient teaching. https://www.wolterskluwer.com/en/expert-insights/ten-tactics-to-improve-patient-teaching.

[35] Peelle JE (2018). Listening effort: how the cognitive consequences of acoustic challenge are reflected in brain and behavior. Ear Hear.

[36] Coleman CK, Muñoz K, Ong CW, Butcher GM, Nelson L, Twohig M (2018). Opportunities for audiologists to use patient-centered communication during hearing device monitoring encounters. Semin Hear.

